# Acute effects of constant torque and constant angle stretching on the muscle and tendon tissue properties

**DOI:** 10.1007/s00421-017-3654-5

**Published:** 2017-06-17

**Authors:** Andreas Konrad, Francesco Budini, Markus Tilp

**Affiliations:** Institute of Sports Science, University of Graz, Mozartgasse 14, 8010 Graz, Austria

**Keywords:** Stiffness, Ultrasound, Passive resistive torque, Maximum voluntary contraction, Range of motion

## Abstract

**Purpose:**

Static stretching induces acute structural changes of the muscle–tendon unit (MTU) that are related to the intensity or duration of stretching. It has been reported that stretching with a constant torque (CT) leads to greater joint range of motion changes than stretching with a constant angle (CA). Whether or not this difference is due to different structural changes of the MTUs of the lower leg and ankle plantar flexors is not known. Therefore, the purpose of this study was to compare the acute effects of single CA and CT stretching on various muscle and tendon mechanical properties.

**Method:**

Seventeen young, healthy volunteers were tested on two separate days using either CT or CA stretching (4 × 30 s each). Before and after stretching, dorsiflexion range of motion (RoM), passive resistive torque (PRT), and maximum voluntary contraction (MVC) were measured with a dynamometer. Ultrasonography of the medial gastrocnemius (GM) muscle–tendon junction (MTJ) displacement allowed us to determine the length changes in the tendon and muscle, respectively, and hence to calculate their stiffness.

**Results:**

Maximum dorsiflexion increased while PRT, muscle–tendon stiffness, and muscle stiffness decreased following both CA and CT stretching. There was a greater increase in RoM following CT stretching compared to CA stretching. Moreover, the decline in PRT was greater during CT stretching compared to CA stretching. As expected, several functional adaptations (RoM, PRT) were different between CT and CA stretching due to the higher intensity of CT stretching. However, no structural differences in the adaptations to the stretching modalities could be detected.

**Conclusion:**

We suggest that the different functional adaptations between CA and CT stretching are the consequence of different adaptations in the perception of stretch and pain.

## Introduction

Stretching is commonly performed before sports participation. There is some evidence that a single stretching exercise, in addition to a warm-up, can reduce the occurrence of muscle strain; however, this does not prevent overuse injuries (McHugh and Cosgrave 2010). Furthermore, some athletes actually avoid stretching pre-exercise due to a possible detrimental effect on the maximal muscle performance. However, Kay and Blazevich (2012) showed in their review article that static stretching interventions of less than 60 s have no disadvantageous effect on maximum performance output. Therefore, especially in sports where a high range of motion (RoM) is required for a good performance, stretching for up to 60 s during the warm-up routine is suggested.

With regard to static stretching, the increased RoM following a single exercise can be explained by a decrease in overall muscle–tendon stiffness (Kay et al. 2015; Konrad et al. 2016) and passive resistive torque (PRT) (Nakamura et al. 2013; Konrad et al. 2016). However, there have been conflicting reports about the effects of acute static stretching on the muscular and tendinous structures of the muscle–tendon unit (MTU). While Kay and Blazevich (2009), Kay et al. (2015), and Konrad et al. (2016) reported a decrease in stiffness of the muscle component, Kubo et al. (2001) and Kato et al. (2010) reported decreased tendon stiffness. These controversial results may be explained by the different stretching durations or intensities utilized.

Concerning stretching intensities/modalities, only a few studies (Herda et al. 2011, 2014; Cabido et al. 2014) have compared the effects of constant angle (CA) stretching (the position is held at a constant joint angle) and constant torque (CT) stretching (where the torque during the stretching is held constant by increasing the joint angle). When a joint is held at a constant angle, passive torque will decrease due to the viscoelastic behavior of the muscle–tendon structure (Magnusson et al. 1996). Hence, it can be assumed that during CT stretching, higher forces act on the MTU compared to CA stretching, and this may explain why muscle–tendon stiffness was reduced following CT only (Herda et al. 2011, 2014). However, other parameters such as maximum voluntary contraction (MVC) (Herda et al. 2011), RoM, and PRT (Herda et al. 2011, 2014) are similarly affected. Cabido et al. (2014) reported that muscle–tendon stiffness and RoM were influenced by both CA and CT stretching; however, greater reductions in muscle–tendon stiffness and greater increases in RoM were observed following CT stretching compared to CA stretching.

In summary, both stretching methods seem to provide different stimuli to the MTU, but it is not yet well understood how these functional differences can be explained on a structural level.

Therefore, the objective of this study was to analyze the acute effects of single CA and CT stretching exercises on the functional and structural parameters of the GM MTU. Furthermore, we attempted to determine the differences between the effects of the two modalities (CA vs. CT). Taking into account reports in the literature, we hypothesized an increase in RoM and adaptations in the MTU (e.g., more compliant muscle tissue) following both CA and CT stretching. However, we expected significantly greater structural changes of the MTU following the CT stretching compared to the CA stretching.

## Methods

### Subjects

Eight healthy female (mean ± SD; 23.3 ± 2.5 years, 167.9 ± 6.3 cm, 58.8 ± 3.9 kg) and nine healthy male (mean ± SD; 24.9 ± 4.2 years, 182.6 ± 6.0 cm, 77.3 ± 6.6 kg) volunteers with no history of lower leg injuries participated in this study. Subjects were informed about the testing procedure, but were not informed about the study’s aims and hypotheses. The study was approved by the local research ethics board, and written informed consent was obtained from all volunteers before the onset of the experimental procedures.

### Experimental design

Participants visited the laboratory for two sessions on different days (2 days to 1 week break in between) at the same time of day. CA and CT stretching trials were performed in a random order. Before and after both stretching procedures (CA and CT), the RoM, PRT, MVC torque, muscle–tendon stiffness, muscle stiffness, and passive and active tendon stiffness of the gastrocnemius medialis muscle (GM) were determined.

### Measures

The temperature in the laboratory was kept constant at around 20.5 °C. Measurements were performed without any warm-up and in the following order: 1. RoM (1-min rest); 2. PRT (1-min rest); 3. MVC (1-min rest); 4. CA or CT stretching for 4 × 30 s; 5. RoM (1-min rest); 6. PRT (1-min rest); 7. MVC.

#### RoM measurement

RoM was determined with an isokinetic dynamometer (CON-TREX MJ, CMV AG, Duebendorf, Switzerland) with the standard setup for ankle joint movement individually adjusted. Subjects were seated with a hip joint angle of 110°, with the foot resting on the dynamometer foot plate and the knee fully extended. Two oblique straps on the upper body and one strap around the thigh were used to secure the participant to the dynamometer and exclude any evasive movement. The foot was fixed barefooted with a strap to the dynamometer foot plate, and the estimated ankle joint center was carefully aligned with the axis of the dynamometer to avoid any heel displacement. Participants were first moved to the neutral ankle joint position in the dynamometer (90°) and were subsequently asked to regulate the motor of the dynamometer with a remote control to get into a dorsiflexion (stretching) position until the point of maximum discomfort was reached. The difference between the maximum dorsiflexion and the neutral position was defined as the dorsiflexion RoM.

#### Passive resistive torque (PRT) measurement

During this measurement, the dynamometer moved the ankle joint from 10° plantar flexion to the individual maximum dorsiflexion RoM, which was previously determined in the RoM measurement. During pilot measurements, we recognized a conditioning effect during the first two passive movements, similar to the active conditioning reported by Maganaris (2003). Therefore, the ankle joint was moved passively for three cycles and measurements were taken during the third cycle to minimize bias caused by the conditioning effect. As in the studies of Kubo et al. (2002) and Mahieu et al. (2009), the velocity of the dynamometer was set at 5°/s to exclude any reflexive muscle activity. Participants were asked to relax during the measurements.

#### Maximum voluntary contraction (MVC) measurement

MVC measurement was performed with the dynamometer at a neutral ankle position (90°). Participants were instructed to perform three isometric MVCs of the plantar flexors for 5 s, with rest periods of at least 1 min between the measurements to avoid any fatigue. The attempt with the highest MVC torque (subtracted from the passive resting torque at this ankle position) value was taken for further analysis.

#### Electromyography (EMG)

Muscular activity was monitored by EMG (myon 320, myon AG, Zurich, Switzerland) during PRT and MVC measurements. After standard skin preparation, surface electrodes (Blue Sensor N, Ambu A/S, Ballerup, Denmark) were placed on the muscle bellies of the GM and the tibialis anterior according to SENIAM recommendations (Hermens et al. 1999). In the PRT measurements, the raw EMG was monitored online to ensure that the subject was relaxed. In the case of an increase in the EMG of the GM or the tibialis anterior being observed, the PRT measurement was repeated.

#### Measurement of elongation of the muscle–tendon structures

A real-time ultrasound apparatus (MyLab 60, Esaote S.p.A., Genova, Italy) with a 10-cm B-mode linear-array probe (LA 923, Esaote S.p.A., Genova, Italy) placed at the MTJ between GM muscle and Achilles tendon was used to obtain longitudinal ultrasound images during PRT and MVC measurements. The ultrasound probe was attached to the lower leg with a custom-built Styrofoam block and secured with elastic bands to prevent any displacement of the probe. During a previous study (Stafilidis and Tilp 2015), we confirmed that this kind of fixation of the ultrasound probe did not lead to any unwanted shifts of the probe during the measurement. To determine the muscle displacement during PRT measurement, the echoes of the MTJ in the ultrasound videos were manually tracked (Kato et al. 2010). Similar to the approach used by other authors (Morse et al. 2008; Kato et al. 2010), the cadaveric regression model of Grieve et al. (1978) was used to obtain the length changes of the MTU of the GM during passive movements. The difference between the overall MTU length change and the displacement of the muscle was defined as the tendon displacement. To determine the tendon displacement during MVC measurement, the echoes of a fascicle insertion at the deep aponeurosis near the MTJ were manually tracked (Kubo et al. 2002; Konrad and Tilp 2014a).

The ultrasound images were recorded at 25 Hz, with an image depth resolution of 74 mm. During PRT and MVC measurements, the videos were synchronized with the other data using a custom-built manual trigger. The videos were cut and digitized in VirtualDub open-source software (version 1.6.19, http://www.virtualdub.org) and analyzed in ImageJ open-source software (version 1.44p, National Institutes of Health, USA). Each video was measured by two investigators, and the mean values of the measurements were used for further analysis of the muscle–tendon structure. Except for the principal investigator, the investigators were not informed of the hypotheses of the study or the group allocation. During the analysis of the PRT measurement, every fifth frame (and for MVC measurement every second frame) was measured by the investigators, corresponding to a time resolution of 0.2 and 0.08 s, respectively.

#### Calculation of muscle/tendon force, passive muscle/tendon stiffness, active tendon stiffness, and muscle–tendon stiffness

The muscle force of the GM was estimated by multiplying the measured torque by the relative contribution of the physiological cross-sectional area (18%) of the GM within the plantar flexor muscles (Kubo et al. 2002; Mahieu et al. 2009), and dividing by the moment arm (MA) of the triceps surae muscle, which was individually measured by tape measure as the distance between the lateral malleolus and the Achilles tendon at rest at neutral ankle position (90°, Konrad and Tilp 2014b). The mean value of the MA was 4.3 cm and the range was 3.5–5.0 cm.

Active tendon stiffness was calculated as the change in the active force divided by the change of the related tendon length during the MVC measurements over a range of force of 50–90% (Kay et al. 2015) at neutral ankle position. The attempt with the highest MVC torque value was taken for active tendon stiffness calculation. Passive tendon stiffness and muscle stiffness were calculated as the change in passive force produced at the last 10° up to maximum dorsiflexion (Magnusson et al. 1997; for the post-trial, a pre-stretching maximum was considered to allow a comparison) divided by the change of the related tendon length/muscle length, respectively. Muscle–tendon stiffness was calculated as the change in PRT produced at the last 10° up to maximum dorsiflexion (Magnusson et al. 1997; for the post-trial, a pre-stretching maximum was considered to allow a comparison) divided by the change of the related joint angle.

### Stretching exercise

The CA and CT stretching exercises were undertaken using the dynamometer, with the starting point at neutral ankle position (90°). During the CA stretching exercise, the subjects were asked to regulate the motor of the dynamometer with a remote control to get into a dorsiflexion (stretching) position corresponding to 95% of the maximum RoM (Cabido et al. 2014) determined during the RoM measurement, with the help of visual feedback. This position was held for 30 s. For the CT stretching, the subjects were asked to regulate the dynamometer to reach the individual PRT corresponding to 95% of the maximum RoM (Cabido et al. 2014). The torque values were provided on a monitor in front of the subjects, and whenever the torque curve decreased by 2 Nm (marked as a line), the volunteer increased the dorsiflexion angle to maintain CT. Similar to the CA stretching exercise, this was done for 30 s. Both the CA and CT stretching procedures were repeated four times, resulting in a total stretch period of 120 s. A rest of 20 s duration in the neutral ankle position was allowed in between stretching bouts. This protocol was chosen because it has been reported that 4 × 30 s of static stretching can decrease MTU stiffness (Ryan et al. 2008).

### Statistical analyses

SPSS (version 20.0, SPSS Inc., Chicago, Illinois) was used for all the statistical analyses. To determine the inter-rater reliability of the muscle–tendon displacement measurements, intraclass correlation coefficients (ICCs) were used. The variables tested were RoM, PRT, MVC, passive tendon stiffness, muscle stiffness, muscle–tendon stiffness, and active tendon stiffness. A Shapiro–Wilk test was used to verify the normal distribution of all the variables. If the data were normally distributed, we performed a two-way repeated-measures ANOVA [factors: time (pre vs. post) and stretching modality (CA vs. CT)]. Otherwise, we performed a Friedman test to test the effects of the stretching protocols (CT and CA). If ANOVA with repeated measures or the Friedman test was significant, we performed a *t* test or a Wilcoxon test (both Bonferroni corrected). To test possible differences between CT and CA stretching protocols, paired *t* tests or Wilcoxon tests of the change between the pre- and post-measurements in any parameter were performed. The alpha level was set at 0.05.

## Results

### Measurement quality

The mean ICCs of the inter-rater test of the ultrasound video analysis were 0.98 and 0.97 for the MTJ displacement during PRT measurement and the MTJ displacement during MVC measurement, respectively. The mean ICC between the baseline values of the CA and CT measurements for all the parameters tested was 0.85.

### Range of motion (RoM)

**Fig. 1:**
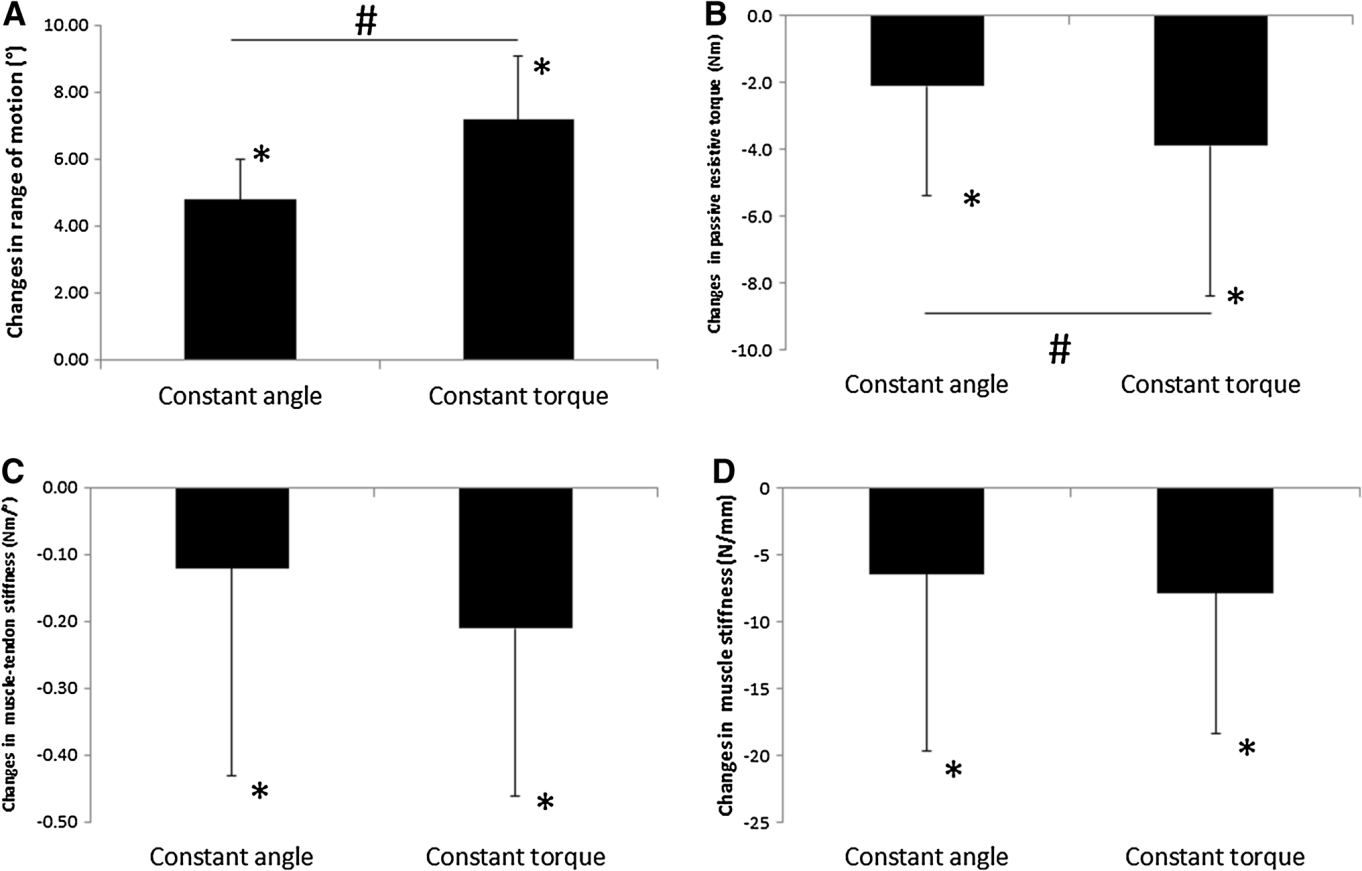
Changes (POST–PRE) in range of motion (**a**); passive resistive torque (**b**); muscle–tendon stiffness (**c**); and muscle stiffness (**d**). ^*^Significant difference between pre- and post-session data; ^#^significant difference between constant angle and constant torque stretching

There was a significant increase in the RoM in both stretching groups (see Table 1; Fig. 1a). Moreover, the CT stretching resulted in a significantly greater increase in RoM compared to the CA stretching (see Table 2; Fig. 1a).

**Table 1:** Maximum dorsiflexion range of motion, as well as passive resistive torque, tendon stiffness, muscle stiffness, and muscle–tendon stiffness during passive measurements

	Constant angle		Constant torque		*P*	*F*	*χ* ^2^
	Pre	Post	Pre	Post			
Range of motion (°)	31.7 ± 6.7	36.6 ± 6.9*	32.8 ± 5.5	40.0 ± 6.1*	0.00^#^	–	41.04
Passive resistive torque (Nm)	26.4 ± 12.5	24.2 ± 10.7*	29.5 ± 13.3	25.6 ± 10.0*	0.00^#^	–	17.26
Passive tendon stiffness (N/mm)	20.9 ± 6.9	22.2 ± 8.5	21.9 ± 10.9	20.0 ± 7.4	0.86	–	0.75
Muscle stiffness (N/mm)	25.4 ± 22.6	19.0 ± 14.0*	26.6 ± 19.1	18.8 ± 11.7*	0.00^#^	–	13.28
Muscle–tendon stiffness (Nm/°)	1.43 ± 0.79	1.31 ± 0.59*	1.54 ± 0.81	1.33 ± 0.61*	0.04^#^	–	8.08
MVC torque (Nm)	113.6 ± 44.5	112.0 ± 43.1	118.1 ± 48.6	114.2 ± 46.2	0.17	1.98	–
Active tendon stiffness (N/mm)	30.8 ± 14.0	31.0 ± 16.2	30.7 ± 10.1	30.2 ± 14.1	0.81	–	0.81

Maximum voluntary contraction torque and active tendon stiffness during maximum voluntary contraction measurements

* Significant difference between pre- and post-session data, mean ± SD

^#^Significant interaction effect (ANOVA) or Friedman test

**Table 2:** Changes (POST–PRE) in maximum dorsiflexion range of motion, as well as passive resistive torque, tendon stiffness, muscle stiffness, and muscle–tendon stiffness during passive measurements

	Constant angle	Constant torque	*P*
	Post–pre	Post–pre	
Range of motion (°)	4.8 ± 1.2	7.2 ± 1.9	0.00*
Passive resistive torque (Nm)	−2.1 ± 3.3	−3.9 ± 4.5	0.01*
Passive tendon stiffness (N/mm)	1.3 ± 7.2	−1.9 ± 6.2	0.20
Muscle stiffness (N/mm)	−6.4 ± 13.2	−7.8 ± 10.5	0.47
Muscle–tendon stiffness (Nm/°)	−0.12 ± 0.31	−0.21 ± 0.25	0.11
MVC torque (Nm)	−1.5 ± 10.5	−3.9 ± 13.7	0.51
Active tendon stiffness (N/mm)	0.2 ± 5.1	−0.5 ± 11.7	0.63

Changes in maximum voluntary contraction torque and active tendon stiffness during maximum voluntary contraction measurements

* Significant difference between constant angle and constant torque stretching, mean ± SD

### Passive resistive torque (PRT) and the related structural muscle–tendon parameters

Significant decreases in PRT, muscle–tendon stiffness, and muscle stiffness were found in both stretching groups (see Table 2; Fig. 1b–d). Furthermore, the decrease in PRT in the CT stretching group was significantly higher than the results in the CA stretching group (see Table 2; Fig. 1b).

### Maximum voluntary contraction (MVC) and active tendon stiffness

There were no significant differences in MVC and active tendon stiffness between the stretching groups (see Table 1). Additionally, no significant differences were detected between CA and CT stretching in MVC torque changes and active tendon stiffness changes (see Table 2).

## Discussion

The purpose of this study was to investigate possible functional and structural differences in the effects of CA and CT stretching exercises on the MTU of the GM. As we anticipated, both the CA and CT stretching induced an increase in RoM and a decrease in PRT, muscle–tendon stiffness, and muscle stiffness. However, in contrast to our expectations, we did not find any differences between the two stretching modalities for structural parameters.

Similar to results from previous studies that used CA (Herda et al. 2011, 2014; Cabido et al. 2014; Konrad et al. 2016) and CT (Herda et al. 2011, 2014; Cabido et al. 2014; Morse et al. 2008; Kato et al. 2010) stretching protocols, RoM increased following both CA and CT stretching. However, when looking at those studies that made a direct comparison of the effects of the two stretching modalities on RoM (Herda et al. 2011, 2014; Cabido et al. 2014), some differences arise. For example, Herda et al. (2011, 2014) found no difference in the effects of CA and CT stretching on the RoM, while both Cabido et al. (2014) and our results showed a significantly greater improvement in RoM following CT stretching. This was further supported by Yeh et al. (2007), who compared CA and CT stretching in stroke patients with ankle hypertonia and also found a higher RoM gain in CT stretching compared to CA stretching. The different results reported by Herda et al. (2011, 2014) might be explained, as already suggested by Cabido et al. (2014), by the adaptation of the stretching position in CA stretching according to the gain in RoM following every single bout. In contrast, the subjects studied by Cabido et al. (2014), as well as those recruited for the present study, received a stretch of 95% of the initial RoM assessment at the CA trial. Therefore, it can be assumed that the subjects in the study of Herda et al. (2011, 2014) received a higher intensity of CA stretching compared to the subjects of the study of Cabido et al. (2014) and the present study. This may explain the lack of difference between the two stretching modalities in the studies of Herda et al. (2011, 2014).

Both PRT and muscle–tendon stiffness decreased following the CA and CT stretching exercises, and this result is in agreement with what is reported in several previous studies (Kay et al. 2009; Nakamura et al. 2013; Konrad et al. 2016). While the decrease in PRT was significantly higher following CT stretching compared to CA stretching, the decrease in muscle–tendon stiffness showed no difference between groups (*P* = 0.11). This discrepancy might be explained by the different approaches used in the calculations. While PRT was determined at the maximum angle of the pre-assessment (in both the pre- and post-measurements), muscle–tendon stiffness was defined as the change in PRT produced over the last 10° up to maximum dorsiflexion. One could therefore assume that the muscle–tendon stiffness might be less sensitive to changes.

Our results are in contrast to the results of Herda et al. (2011, 2014), who reported a decrease in muscle–tendon stiffness following CT stretching, but not following CA stretching. Similarly, Cabido et al. (2014) reported a decrease in muscle–tendon stiffness in both stretching modalities, but the decrease was higher in the CT stretching exercise. A possible explanation for the different results might be the different muscles investigated. While the above-mentioned studies explored the leg extensors, in the current study, we investigated the plantar flexor muscles. Different agonist and antagonist muscles of the different muscle groups could have affected the results.

Furthermore, Herda et al. (2011, 2014) and Cabido et al. (2014) included only male subjects in their studies, while female subjects were also included in the current study. However, in our data, a post hoc analysis (data not shown) showed only weak evidence of differences in the effects of CA and CT stretching between males and females. Out of 16 variables, we found only one significant difference from CA stretching, in passive tendon stiffness (results post–pre; men/women (mean ± SD; 5.14 ± 5.71/−3.58 ± 6.04 N/mm), *P* = 0.01).

In addition to several parameters of the muscle–tendon function (RoM, PRT, MVC, and muscle–tendon stiffness), we also investigated the muscle–tendon structure, namely, muscle stiffness and tendon stiffness. As in previous studies (Kay and Blazevich 2009; Kay et al. 2015; Konrad et al. 2016), we observed a decrease in muscle stiffness but not in tendon stiffness following both static stretching exercises (CA and CT). However, others have reported a decrease in tendon stiffness (Kubo et al. 2001; Kato et al. 2010) with no changes in muscle stiffness (Kato et al. 2010) following a single static stretch. Possible reasons for these controversial results might be found in the different stretch durations, which we already discussed in Konrad et al. (2016).

We hypothesized that the higher gain in RoM observed after CT stretching compared to that observed after CA stretching would have been accompanied by a greater loss of muscle stiffness or tendon stiffness. However, we did not observe any difference between the changes in muscle and tendon stiffness. Comparing the absolute value changes of the muscle stiffness for both stretching modalities, there was a tendency for a greater decrease in muscle stiffness in CT stretching (−7.8 N/mm) compared to CA stretching (−6.4 N/mm) stretching. Moreover, both active and passive tendon stiffness showed a slight decrease in CT stretching (active, −0.5 N/mm; passive, −1.9 N/mm) and a slight increase in CA stretching (active, 0.2 N/mm; passive, 1.3 N/mm) (Table 2). Although the single results did not reach statistical significance, a combination of both might explain the higher gain in RoM for CT stretching compared to CA stretching on a structural level.

A further reason for the differences in RoM could be dissimilar adaptations in the perception of stretch and pain, or stretch tolerance (Halbertsma et al. 1996; Magnusson et al. 1996) between the stretching methods. One could assume that due to the higher intensity of the CT stretching compared to the CA stretching, the stretch and pain tolerance increased more based on more pronounced adaptations of nociceptive nerve endings. Our data showed an increased PRT following both stretching modalities (CA: 5.62 ± 5.32 [*P* = 0.00]; CT: 6.79 ± 6.08 [*P* = 0.00]) at the end RoM, indicating an increase in stretch tolerance. Although the amount of increased PRT was higher for CT stretching than CA stretching, this did not reach statistical significance (*P* = 0.23).

Another possible explanation can be proposed on the basis of a stretch-induced alteration of neuromuscular activity. It is known that slow stretches provoke tonic stretch reflexes (Matthews 1964), which, similar to phasic stretch reflexes, produce a contraction of the muscle being stretched. Such a contraction will certainly increase the “passive torque” value and possibly also affect the “RoM” parameter (PNF-like stretching). It could therefore be suggested that CA and CT stretching affect the tonic stretch reflex in different ways. It has been demonstrated that stretching decreases muscle spindle sensitivity due to formation of slack in the intrafusal fibers (Proske et al. 1993). It is therefore conceivable that CT stretching, being more intense than CA stretching, also has a greater effect on muscle spindles.

A further explanation for the lack of a greater loss of muscle stiffness or tendon stiffness in CT stretching compared to CA stretching might be found in the other muscles of the lower leg. We cannot rule out stiffness changes of other plantar flexor muscles (gastrocnemius lateralis and/or soleus), which could explain the greater improvement in RoM following CT stretching compared to CA stretching.

Although we undertook this study as objectively as possible, there are still some limitations we have to mention. Firstly, the investigators were not blinded to the intervention, and experimenter bias in the results cannot be completely excluded. However, the inter-rater reliability scored high (mean ICC: 0.98 for the PRT measurement and 0.97 for the MVC measurement), which indicates high objectivity. As a second potential limitation, the method of measuring the MA of the ankle joint in vivo was quite simple, and we assumed a constant MA during contraction and passive ankle movement. However, the values obtained in this study were very similar to others obtained using magnetic resonance imaging (Rugg et al. 1990) or ultrasound (Lee and Piazza 2009). Furthermore, possible errors would be the same in both stretching modalities, and would therefore not alter the main results of the study. However, we cannot rule out different changes in MA following CA stretching compared to CT stretching. Thirdly, our stiffness measurements of the Achilles tendon included the superficial soleus aponeurosis, and the free Achilles tendon stiffness response may be different to the response of the aponeurotic portion of the Achilles tendon.

## Conclusion

We conclude that both a single CA and CT stretching protocol increase the ankle RoM. The change in RoM can be explained by the more compliant GM muscle tissue, accompanied by a decrease in PRT. Although we found no differences in the effects of muscle and tendon stiffness between CA and CT stretching, we would recommend CT stretching in sports practice for higher gains in RoM compared to CA stretching.

## Abbreviations

ANOVA: Analysis of variance
CA: Constant angle
CT: Constant torque
EMG: Electromyography
GM: Gastrocnemius medialis
ICC: Intraclass correlation coefficient
MA: Moment arm
MTJ: Muscle–tendon junction
MTU: Muscle–tendon unit
MVC: Maximum voluntary contraction
PRT: Passive resistive torque
RoM: Range of motion
SD: Standard deviation
